# Supplementary material for: Evolutionary Dynamics of FLC-like MADS-Box Genes in Brassicaceae
Source: Plants (Basel). 2023 Sep 15;12(18):3281. doi: 10.3390/plants12183281 (PMC10536770; doi:10.3390/plants12183281)

Exon-intron structures of *MAF4/5*-like genes

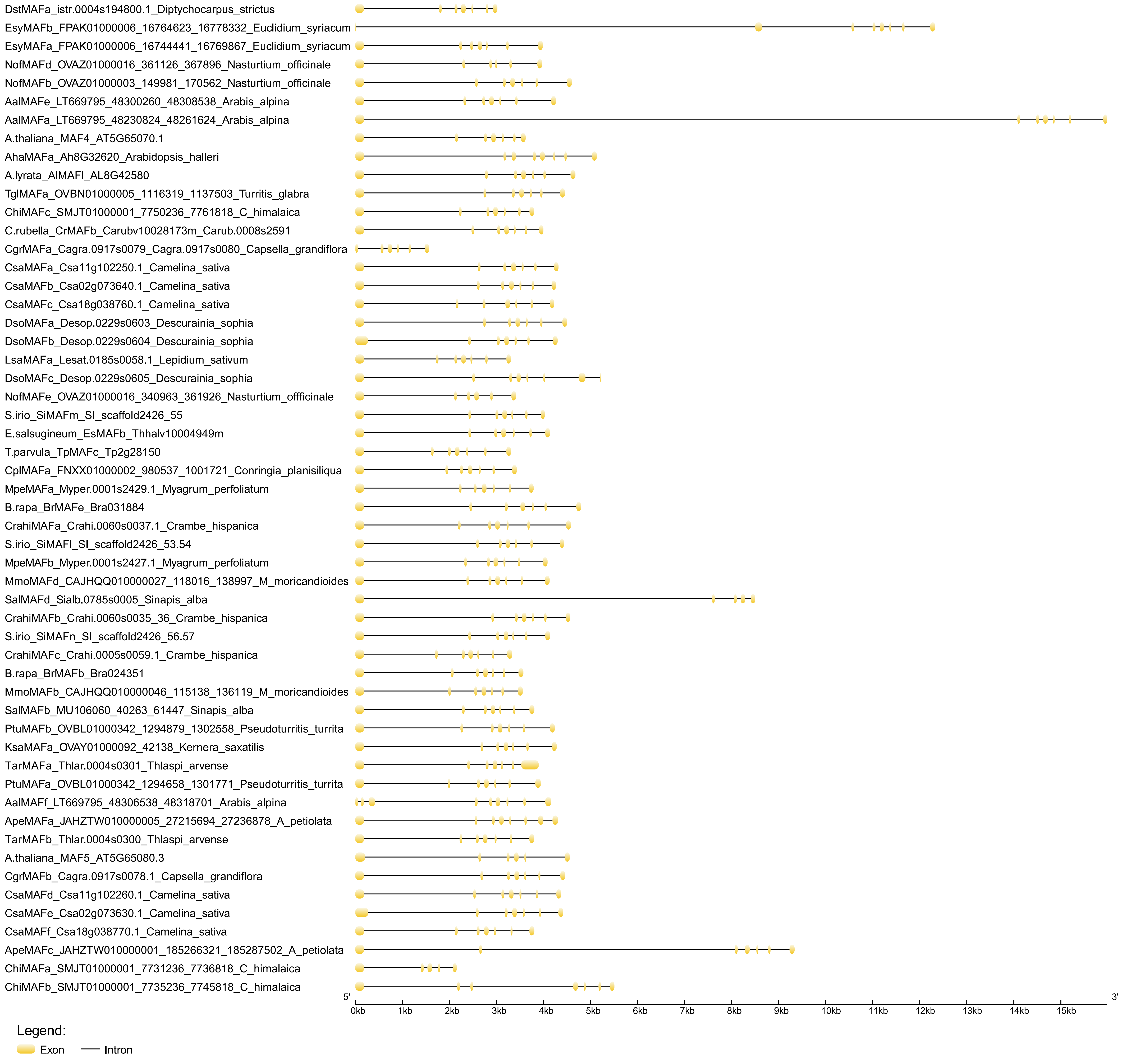

Exon-intron structures of *MAF1/2/3*-like genes

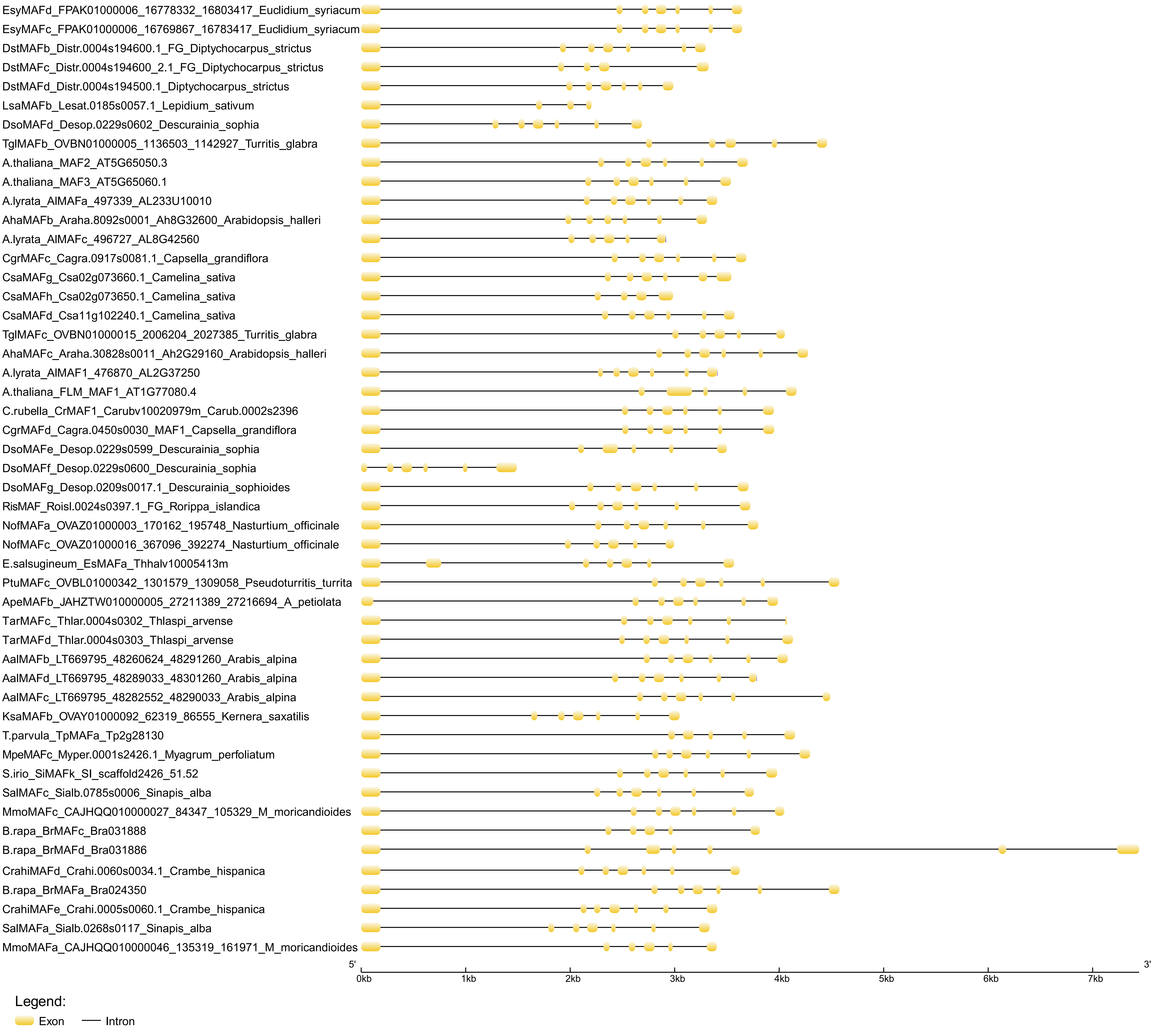

Exon-intron structures of *FLC*-like genes *s.str.*

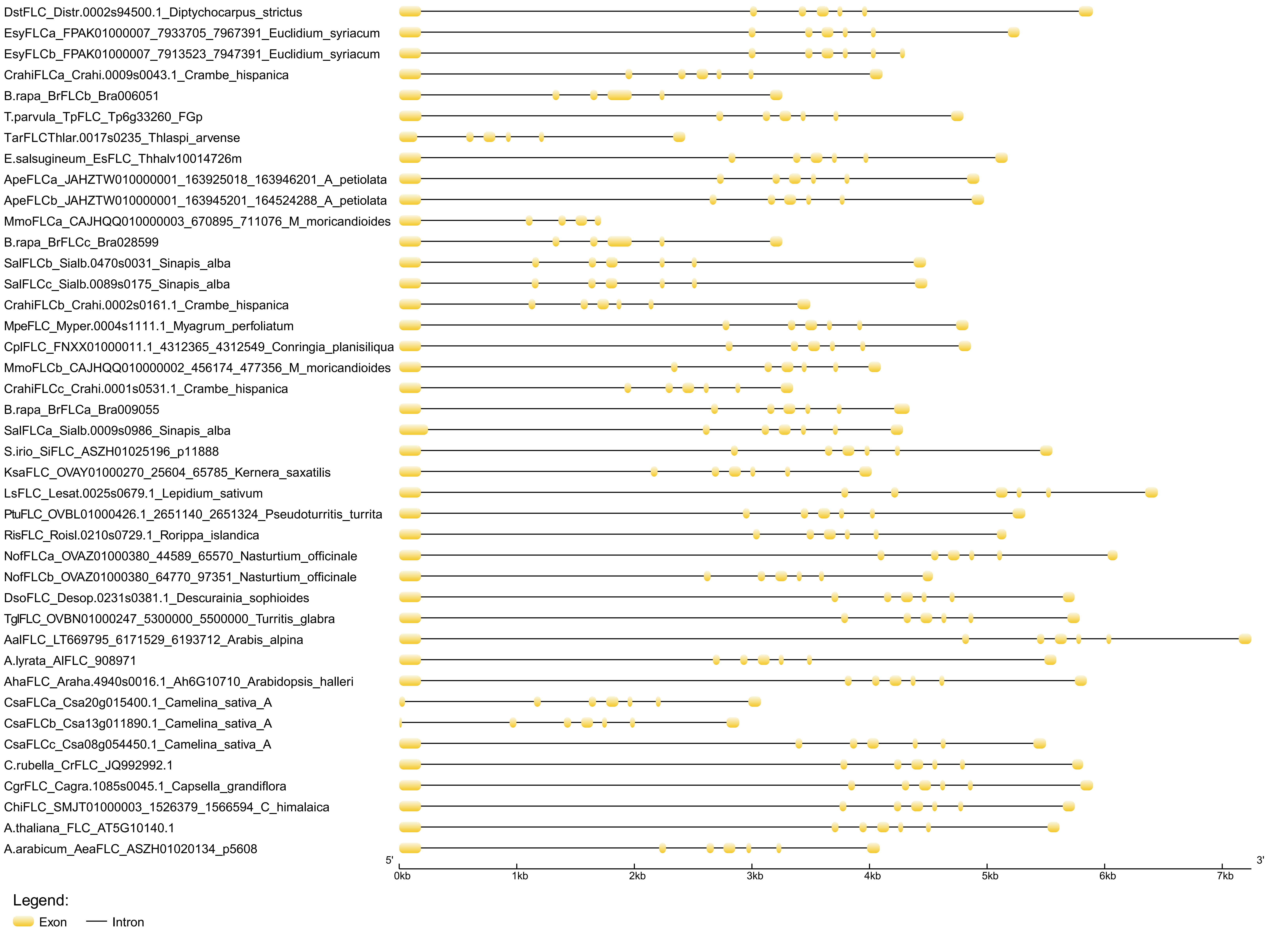

Supplement: Supplementary file 1 [file plants-12-03281-s001.zip › FigureS1_Exon_intron_structures_FLC_like_genes_v2.pdf]
