# Supplementary material for: Evolutionary Dynamics of FLC-like MADS-Box Genes in Brassicaceae
Source: Plants (Basel). 2023 Sep 15;12(18):3281. doi: 10.3390/plants12183281 (PMC10536770; doi:10.3390/plants12183281)
Supplement: Supplementary file 1 [file plants-12-03281-s001.zip › TableS1_Datasource.pdf]

Table S1: Source of data for Brassicaceae species in which *FLC*-like genes *s.l.* were identified.

**Species**	**Source of data**
Arabidopsis thaliana	Theißen et al., 2018
Arabidopsis lyrata	Theißen et al., 2018
Arabidopsis halleri	BRAD/Phytozome
Capsella rubella	Theißen et al., 2018
Capsella grandiflora	BRAD/Phytozome
Camelina sativa	BRAD
Turritis glabra	NCBI genomes
Crucihimalaya himalaica	NCBI genomes
Lepidium sativum	Phytozome
Nasturtium officinale	NCBI genomes
Rorippa islandica	Phytozome
Descurainia sophia	BRAD/Phytozome
Schrenkiella parvula	Theißen et al., 2018
Eutrema salsugineum	Theißen et al., 2018
Thlaspi arvense	BRAD/Phytozome
Alliaria petiolata	NCBI genomes
Sisymbrium irio	Theißen et al., 2018
Brassica rapa	Theißen et al., 2018
Sinapis alba	NCBI genomes
Moricandia moricandioides	NCBI genomes
Crambe hispanica	Phytozome
Pseudoturritis turrita	NCBI genomes
Arabis alpina	NCBI genomes
Kernera saxatilis	NCBI genomes
Conringia planisiliqua	NCBI genomes
Myagrum perfoliatum	Phytozome
Euclidium syriacum	NCBI genomes
Diptychocarpus strictus	Phytozome
Aethionema arabicum	Theißen et al., 2018
Tarenaya hassleriana	Theißen et al., 2018

Theißen, G.; Rumpler, F.; Gramzow, L. Array of MADS-Box Genes: Facilitator for Rapid Adaptation? *Trends Plant Sci* **2018**, *23*, 563-576, doi:10.1016/j.tplants.2018.04.008.
